# Inhibition of IL‐17 prevents the progression of traumatic heterotopic ossification

**DOI:** 10.1111/jcmm.16617

**Published:** 2021-06-29

**Authors:** Bing Tu, Bo Yu, Wei Wang, Juehong Li, Feng Yuan, Jing Zhu, Cunyi Fan

**Affiliations:** ^1^ Department of Orthopaedics Shanghai Jiaotong University Affiliated Sixth People’s Hospital Shanghai China; ^2^ Department of Sports Medicine Shanghai Jiao Tong University Affiliated Sixth People's Hospital Shanghai China; ^3^ Department of Orthopaedics Affiliated Hospital of Shandong University of Traditional Chinese Medicine Jinan China; ^4^ Department of Anatomy, School of Basic Medicine Shanghai University of Traditional Chinese Medicine Shanghai China

**Keywords:** heterotopic ossification, IL‐17, trauma, β‐catenin

## Abstract

Traumatic heterotopic ossification (HO) is the abnormal formation of bone in soft tissues as a consequence of injury. However, the pathological mechanisms leading to traumatic HO remain unknown. Here, we report that aberrant expression of IL‐17 promotes traumatic HO formation by activating β‐catenin signalling in mouse model. We found that elevated IL‐17 and β‐catenin levels are correlated with a high degree of HO formation in specimens from patients and HO animals. We also show that IL‐17 initiates and promotes HO progression in mice. Local injection of an IL‐17 neutralizing antibody attenuates ectopic bone formation in a traumatic mouse model. IL‐17 enhances the osteoblastic differentiation of mesenchymal stem cells (MSCs) by activating β‐catenin signalling. Moreover, inhibition of IL‐17R or β‐catenin signalling by neutralizing antibodies or drugs prevents the osteogenic differentiation of isolated MSCs and decreases HO formation in mouse models. Together, our study identifies a novel role for active IL‐17 as the inducer and promoter of ectopic bone formation and suggests that IL‐17 inhibition might be a potential therapeutic target in traumatic HO.

## 1 INTRODUCTION

Heterotopic ossification (HO) is a clinical pathology characterized by the ectopic formation of bone within soft tissues. Trauma‐induced HO develops as a common post‐operative complication after orthopaedic surgeries (eg hip arthroplasty), blast injuries, skeletal trauma, severe burns and nervous system injuries.^1^, ^2^ Trauma‐induced HO is initiated by local connective tissue destruction and requires inflammation. This inflammatory microenvironment activates a resident pool of interstitial progenitors that aberrantly undergo chondrogenesis and further ectopic bone formation.^3^ Following trauma and inflammation, ectopic bones are formed in soft tissue through endochondral ossification.^4^ However, the pathological mechanism of trauma‐induced HO is not clear. Clinical therapy is now limited to anti‐inflammatory drugs, radiation or surgical excision of the already formed bone, which is associated with a high recurrence rate.^5^, ^6^ Previous studies have shown that inflammation plays an important role in trauma‐induced HO and FOP.^7^ Many cytokines in the inflammatory microenvironment can activate the progenitors, induce chondrogenesis/osteogenesis and lead to bone formation.^8^, ^9^

IL‐17 can be produced by many types of cells, including T helper 17 (Th17) cells, CD8+ T cells, innate lymphoid (ILC3s) cells and natural killer T cells.^10^ The effects of IL‐17 on inflammation and bone are largely unknown. Previous studies have provided evidence of a catabolic function for IL‐17 in bone homeostasis in that IL‐17 induces the differentiation of osteoclasts, thereby explaining the development of bone resorption in patients with rheumatoid arthritis (RA).^11^, ^12^ In support of this evidence, inhibition of IL‐17 with neutralizing antibodies reduces its bone erosion effects.^13^ In addition to bone destruction, recent studies have provided evidence that IL‐17 promotes osteoblast differentiation and subsequent bone formation. Some studies suggest that IL‐17 induces the differentiation of mesenchymal stem cells (MSCs) into osteoblasts.^14^, ^15^ Clinical trials performed with IL‐17 blocking monoclonal antibodies have clearly shown that IL‐17 inhibition is an effective treatment for ankylosing spondylitis (AS), a disorder characterized by new bone formation.^16^, ^17^ Notably, recent studies have shown that IL‐17 promoted the osteoblast differentiation of isolated MSCs, whereas an inhibitory effect of IL‐17 on whole bone was observed.^18^ Taken together, these evidence indicate that the effect of IL‐17 is complex in that it can promote and inhibit bone formation. The effect of IL‐17 on bone may depend on different diseases and the interactions with other cells.

The Wnt/β‐catenin signalling pathway has been proven to play a critical role in promoting osteogenic differentiation of MSCs. In addition, β‐catenin is a central molecule that is necessary to maintain bone homeostasis and mechanotransduction through the maintenance of osteocyte viability.^19^ The Wnt/β‐catenin signalling pathway is essential for bone mass maintenance by regulating the activity of osteoblasts directly. β‐Catenin conditional activation mice also showed OA‐like changes in the knee joint.^20^ Based on these observations, we hypothesize that IL‐17 released during the inflammation phase of traumatic HO activates β‐catenin signalling, leading to overgrowth of bony tissue during disease progression. The key questions that need to be addressed are the molecular mechanisms involved in the inflammation‐regulated HO process.

We have previously demonstrated that activated β‐catenin is associated with traumatic HO formation.^21^ In this study, we show that IL‐17 is highly induced in traumatic HO and promotes bone formation via β‐catenin signalling. Furthermore, we reveal that IL‐17 enhanced the osteoblast differentiation of MSCs isolated from the mice, which function as the crucial promoter in traumatic HO. Inhibition of IL‐17 activity effectively attenuated traumatic HO progression in the mouse model.

## 2 MATERIALS AND METHODS

### 2.1 Patients and specimens

The study was approved by the ethics committee of Shanghai Jiao Tong University Affiliated Sixth People's Hospital, and written informed consent was obtained from the patients or their legal guardians. Traumatic HO was identified by X‐ray and CT radiography from 30 patients (18 males and 12 females, previously healthy, age ranging from 20 to 57 years) who had previously suffered an elbow fracture that was treated by external or internal fixation (within 1 week from the initial injury). These patients returned for surgical resection of HO. Osteogenesis (15 patients, 2‐3 months after injury) or maturation stage HO (15 patients, 8 months after injury) was defined based on the time since their injury occurred. Healthy muscles were collected from 8 patients who underwent traumatic forearm amputation. The muscles were used as baseline controls. Blood samples (5 mL per person) were collected 1 day before the clinical surgery. Blood samples from 10 healthy individuals were used as baseline controls. All the samples were processed immediately to collect serum, which was then stored in a −80°C freezer. The serum specimens were processed for ELISA.

### 2.2 Mice

Male 6‐week‐old BALB/c mice were anaesthetized with an intraperitoneal injection of pentobarbital sodium. A 1‐cm longitudinal skin incision was made on the lateral aspect of the Achilles tendon to expose its full length. The Achilles tendon was then divided sharply at its midpoint with a surgical knife. For the sham operation, the incision was made through the skin without touching the Achilles tendon. The incised skin was closed with absorbable sutures. The mice were injected with IL‐17 antibody (5 mg/kg) twice per week. Mice were maintained under specific pathogen‐free conditions, and all experiments were approved by the Animal Research Committee of Shanghai Jiao Tong University Affiliated Sixth People's Hospital.

### 2.3 Histology

Mice were killed by carbon dioxide (CO_2_) inhalation and perfusion fixed with 10% buffered formalin via the left ventricle for 5 minutes. Then, the ankles with Achilles tendons were dissected and fixed in 4% paraformaldehyde for 24 hours. All of these specimens were decalcified in a 10% EDTA solution for 1 month, embedded in paraffin and cut into 5‐μm sections for staining.

Trap staining was performed following the manufacturer's protocol (Sigma‐Aldrich, 387A‐1KT), followed by counterstaining with Methyl Green (Sigma‐Aldrich, M884).

Sections were stained with 0.1% Safranin O and 0.02% Fast Green (Sigma‐Aldrich) according to the manufacturer's instructions.

Immunohistochemical staining was carried out with primary antibodies against IL‐17, IL‐17R (Abcam, Cat No. ab11370) and β‐catenin (Cell Signaling Technology, Cat No. 4370) with a 1:1000 dilution of an appropriate secondary antibody. Protein expression was visualized with a DakoCytomation EnVision staining kit. The mean density of the positive area was measured by Image‐Pro Plus 6.0 (IPP) image analysis software. Three random slides were selected, and five random fields of images per sample were taken.

### 2.4 μ‐CT

Achilles tendons and total hindlimbs from mice were fixed overnight in 4% paraformaldehyde. μ‐CT was performed using a SkyScan with a voltage of 60 kV and a resolution of 18 μm, according to standard nomenclature. The region of interest (ROI) was set as the entire tibia to ensure that all the heterotopic bone was included within the ROI. Three‐dimensional (3D) images were reconstructed using NRecon, and HO bone volumes were analysed by CTAn software.

### 2.5 Serum IL‐17 analysis

The concentration of IL‐17 in the serum was determined by the IL‐17 Quantikine ELISA Kit (human: D1700; mouse: M1700. R&D Systems) following the manufacturer's instructions.

### 2.6 Isolation of cells from mice

Bone marrow cells were harvested from the femur by flushing with PBS and seeded at a density of 1 × 10^6^ into 10‐cm culture dishes (Corning, NY, USA) at 37°C and 5% CO_2_. Non‐adherent cells were discarded after 24 hours, and attached cells were cultured in Dulbecco's modified Eagle's medium (DMEM; HyClone) with 10% foetal bovine serum (FBS).

### 2.7 In vitro differentiation of osteoblasts

Cells were resuspended in Dulbecco's modified Eagle's medium (DMEM; HyClone) with 10% foetal bovine serum (FBS) and seeded in 3‐cm culture dishes. After confluency was reached, osteogenic differentiation was induced by recombinant mouse BMP‐2 (500 ng/mL), and IL‐17 (50 ng/mL) was added coincidentally with osteogenesis.

### 2.8 ALP and Alizarin red S staining

For ALP staining, cells were stained with 5‐bromo‐4‐chloro‐3‐indolyl‐phosphate/nitro‐blue tetrazolium solution (Sigma‐Aldrich) for 45 minutes at 37°C to visualize ALP activity. For Alizarin red S staining, cells were fixed in 4% paraformaldehyde for 10 minutes and rinsed 3 times with deionized water. The cells were then stained with 40 mmol/L Alizarin red S (Sigma), pH 4.0, for 10 minutes. Finally, the cells were rinsed 3 times with deionized water with gentle agitation.

### 2.9 Quantitative analysis of ALP activity

Cells were washed twice with PBS and solubilized with lysis buffer (10 mmol/L Tris‐HCl [pH 7.5], 150 mmol/L NaCl, complete protease inhibitor, and 1% NP‐40). ALP activity was assayed using p‐nitrophenylphosphate (Sigma‐Aldrich) as a substrate. The protein content was measured using the BCA Protein Assay kit (Thermo Scientific) according to the manufacturer's instructions. The ALP activity was expressed as Sigma unit/min/mg of protein.

### 2.10 Quantitative analysis of mineralization

The calcium deposits from osteoblast cells were washed 3 times with PBS and incubated for 24 hours at 4°C in 0.5 M HCl. Then, the calcium content in the HCl supernatants was measured using the Calcium Colorimetric Assay Kit (BioVision).

### 2.11 RNA isolation and real‐time PCR

Total RNA from the cells was prepared with TRIzol Reagent (Invitrogen). Complementary DNAs (cDNAs) were synthesized using the iScript cDNA Synthesis Kit (Bio‐Rad) according to the manufacturer's instructions. Quantitative real‐time PCR was carried out using the ABI 7500 Sequence Detection System and SYBR Premix Ex Taq (Takara, Japan). The following primers were used: Runx2: forward 5′‐CCGCC TCAGTGATTTAGGGC −3′, reverse 5′‐ GGGTCTGTAATCTGACTCTG TCC −3′. ALP: forward 5′‐TGAGGGTGTGGCTTACCAG‐3′, reverse 5′‐ GATGGACGTGTAGGCTTTGCT‐3′. OCN: forward 5′‐CCTCAC ACTCCTCGCCCTATT‐3′, reverse 5′‐CCCTCCTGCTTGGACACAAA‐3′. GAPDH：forward 5′‐ATGGGGAAGGTGAAGGTCG‐3′ reverse 5′‐GGGG TCATTGATGGCAACAATA‐3′. Sp7: forward 5′‐ATGGCGTCCTCTCTGCTTG‐3′, reverse 5′‐TGAAAGGTCAGCGTATGGCTT‐3′. All of the reactions were performed in triplicate.

### 2.12 Western blot

The cells were washed in ice‐cold PBS before lysis with a cell lysis buffer (Cell Signaling Technology). All samples were clarified by centrifugation at 12 000 rpm for 10 minutes at 4°C. Then, the protein concentrations were determined using the BCA Protein Assay kit (Thermo Scientific). Equal amounts of total protein lysates were separated by SDS‐PAGE, and bands were transferred to a nitrocellulose membrane. Membranes were probed with specific antibodies to β‐catenin and β‐actin (Cell Signaling Technology) and then reprobed with appropriate secondary antibodies labelled with IR dyes. Bound antibodies were detected with an Odyssey Infrared Imaging System (LI‐COR Biosciences). Densitometric analysis of the protein bands was performed with Image‐Pro Plus 4.5 software (Media Cybernetics).

### 2.13 Statistical analyses

The data are represented as the means ± standard deviation (SD). Comparisons between groups were performed using Student's *t* test, and one‐way ANOVA was used for multiple comparisons. All of the experiments were repeated at least 3 times, and representative experiments are shown. Differences were considered significant at *P* < .05.

## 3 RESULTS

### 3.1 IL‐17 is overexpressed in patients with HO

**FIGURE 1:**
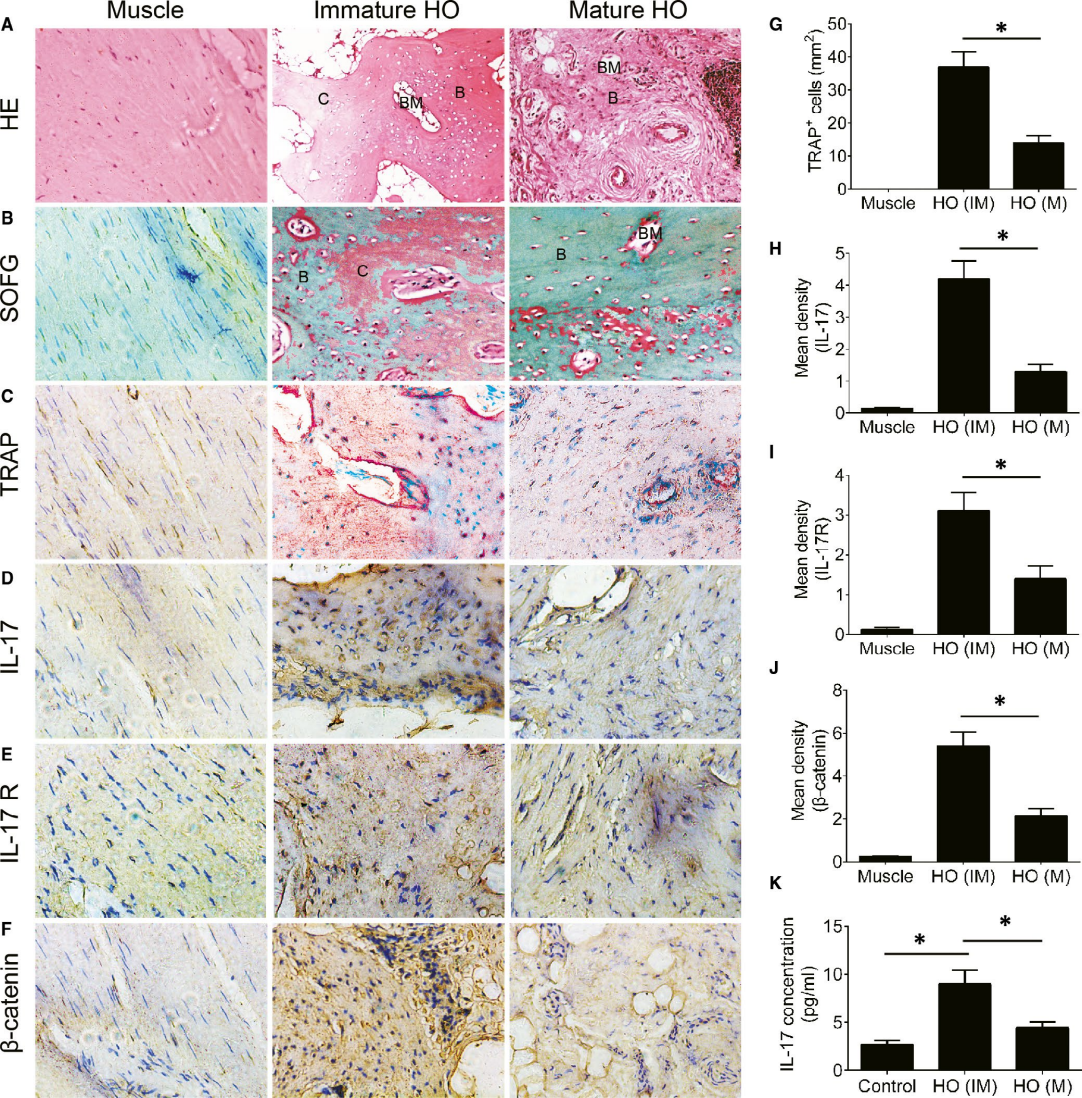
IL‐17 is overexpressed in human HO. (A) H&E staining of normal muscle, immature HO and mature HO from elbow fracture patients. C cartilage, B bone, BM bone marrow. (B) Safranin O and Fast Green (SOFG) staining of normal muscle and HO. Proteoglycan was stained red, and bone tissue was stained green. (C) TRAP^+^ cells (red) in normal muscle and HO. Immunohistochemistry staining of (D) IL‐17, (E) IL‐17R and (F) β‐catenin. (G) Quantitative analysis of TRAP^+^ cells. Histomorphometry analysis of (H) IL‐17, (I) IL‐17R and (J) β‐catenin expression. (K) IL‐17 levels in serum from the patients were determined by ELISA. n = 15 per group. The data are shown as the means ± SD. *P < .05

Patients with HO after elbow fracture were identified by X‐ray imaging. The surgical HO specimens were collected at the immature stage (2‐3 months after initial injury) and maturation stage (>8 months after initial injury).^22^ H&E and Safranin O and Fast Green (SOFG) staining showed a thick layer of cartilage adjacent to the bone at the immature stage. However, we observed a larger cancellous bone and marrow and a thinner cartilage layer at the maturation stage (Figure 1A,B). We next tested osteoclast activity. The TRAP staining results showed that the number of TRAP^+^ cells was significantly increased at the immature stage and decreased at the maturation stage (Figure 1C,G). The expression of IL‐17 and IL‐17R was significantly elevated at the immature stage and decreased at the maturation stage (Figure 1D,H,E,I). Furthermore, immunohistochemistry staining showed that the expression of β‐catenin was significantly increased at the immature stage and decreased at the maturation stage (Figure 1F,J). IL‐17 concentrations in the serum of patients with HO were examined by ELISA, and significantly elevated IL‐17 levels were observed in patients with HO compared with healthy controls. The IL‐17 level was decreased at the maturation stage compared with the immature stage (Figure 1K).

### 3.2 IL‐17/β‐catenin signalling is activated in a traumatic HO mouse model

**FIGURE 2:**
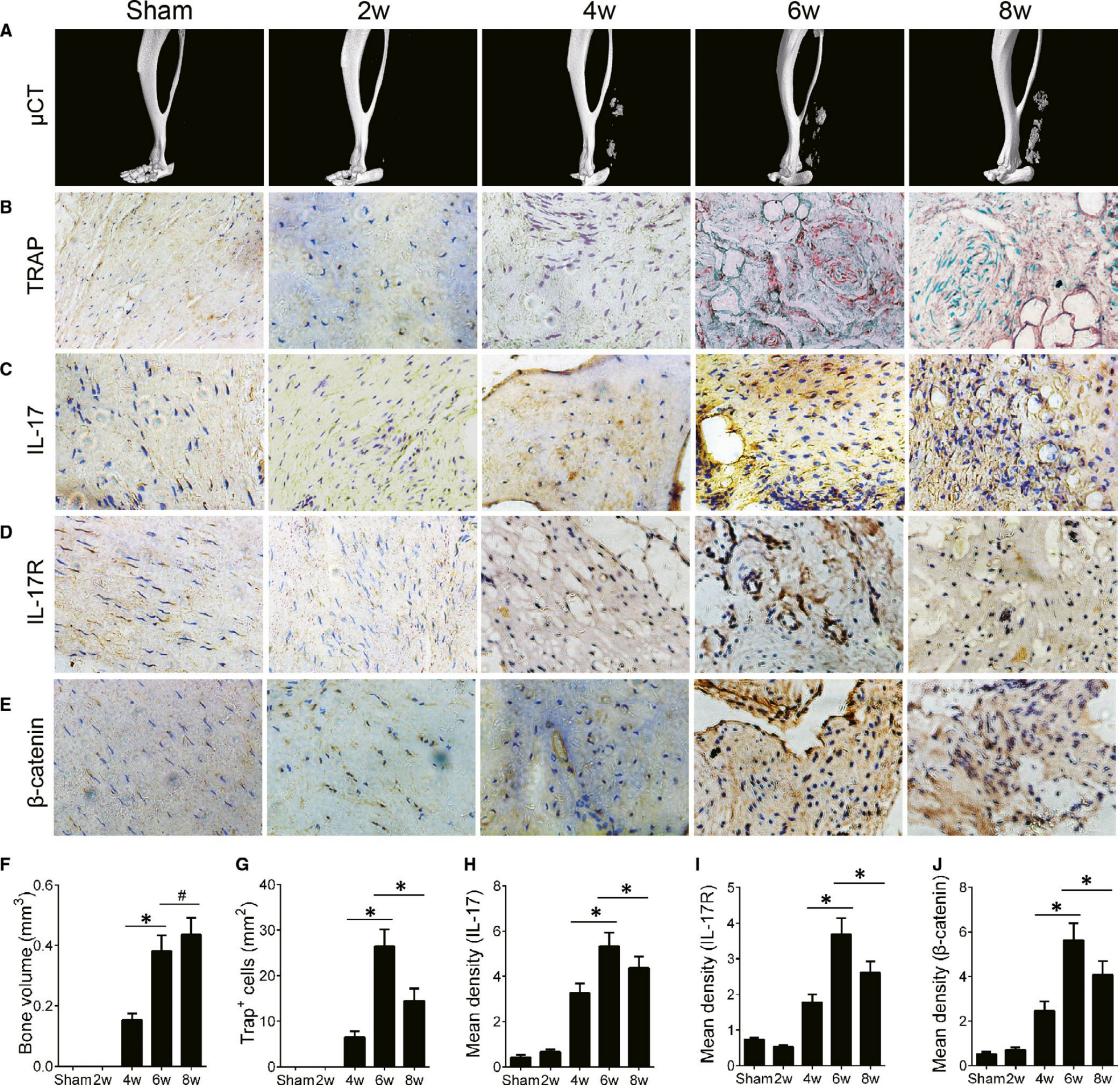
Activated IL‐17/β‐catenin signalling is associated with HO formation in a mouse model. The mice underwent a tenotomy to generate a traumatic HO model. (A) μCT images of HO in the Achilles tendon after sham operation or Achilles tenotomy at 2, 4, 6 and 8 wk. (B) TRAP^+^ cells in HO tissue. Immunohistochemical staining of (C) IL‐17, (D) IL‐17R and (E) β‐catenin. (F) Quantitative analysis of HO volume by μCT. (G) Quantitative analysis of TRAP^+^ cells. Histomorphometry analysis of (H) IL‐17, (I) IL‐17R and (J) β‐catenin expression. All data are shown as the mean ± SD. n = 8 per group. *P < .05

To explore the role of IL‐17 in HO progression, we used a traumatic HO mouse model of the Achilles tendon. Heterotopic bone was formed at the injury site at 4 weeks and grew up to 8 weeks after initial Achilles tendon resection (Figure 2A,F). Immature bone was observed at 6 weeks and developed cancellous bone with marrow at 8 weeks (Figure 2B). Similar to human HO, TRAP staining showed that the number of TRAP^+^ cells in the ectopic bone increased at the initial stage (week 4) after tenotomy and decreased later at week 8. Continuous osteoclast bone resorption produced a large bone marrow cavity (Figure 2B,G). The immunohistochemical staining showed that the expression of IL‐17 (Figure 2C,H), IL‐17R (Figure 2D,I) and β‐catenin (Figure 2E,J) increased at week 4, peaked at week 6 and decreased at week 8. Taken together, the Achilles tendon HO mouse model shows a similar mechanism as that observed in human HO specimens, suggesting that a high level of IL‐17 may contribute to the pathogenesis of HO.

### 3.3 IL‐17 antibody treatment decreases the formation of traumatic HO

**FIGURE 3:**
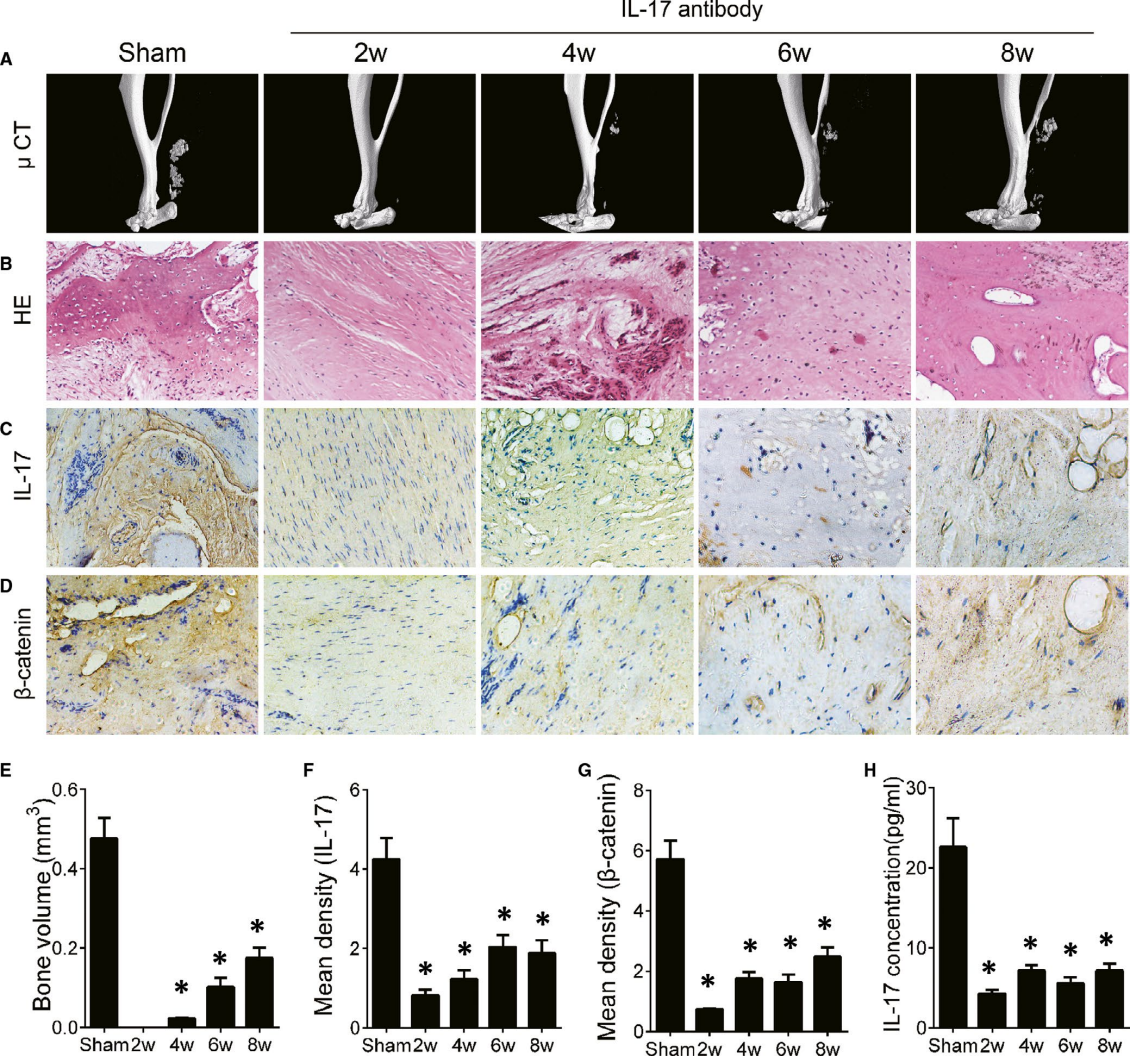
IL‐17 antibody treatment decreases the formation of traumatic HO. The traumatic HO mice were injected with 5 mg/kg bodyweight of the IL‐17 neutralizing antibody at the Achilles tendon twice a week from day 0. (A) μCT images of HO in the Achilles tendon after sham operation or Achilles tenotomy at 2, 4, 6 and 8 wk. (B) H&E staining of ectopic bone formation in Achilles tendons. Immunohistochemical staining of (C) IL‐17 and (D) β‐catenin. (E) Quantitative analysis of HO volume by μCT. Quantification of (F) IL‐17 and (G) β‐catenin expression in HO. (H) IL‐17 levels in mouse serum were determined by ELISA at 2, 4, 6 and 8 wk. All data are shown as the mean ± SD. n = 8 per group. *P < .05

We then examined whether inhibition of IL‐17 activity attenuates HO progression. An IL‐17 neutralizing antibody was injected into the Achilles tendon tenotomy–induced HO mice at the lesion site three times per week. The mice were killed under anaesthesia at 2, 4, 6 and 8 weeks after initial Achilles tenotomy. HO formation was significantly mitigated with injection of IL‐17 neutralizing antibody compared with the control antibody‐treated mice (Figure 3A,B,E). Immunostaining showed that IL‐17 expression was significantly decreased in HO tissue from mice injected with IL‐17 neutralizing antibody compared with control antibody‐injected mice (Figure 3C,F). In addition, the expression of β‐catenin was decreased after the injection of IL‐17 antibody (Figure 3D,G). Similar results were obtained regarding the active IL‐17 concentration in mouse serum (Figure 3H). These data demonstrate that inhibition of IL‐17 signalling activity efficiently attenuates HO progression.

### 3.4 Inhibition of β‐catenin suppresses the formation of traumatic HO

**FIGURE 4:**
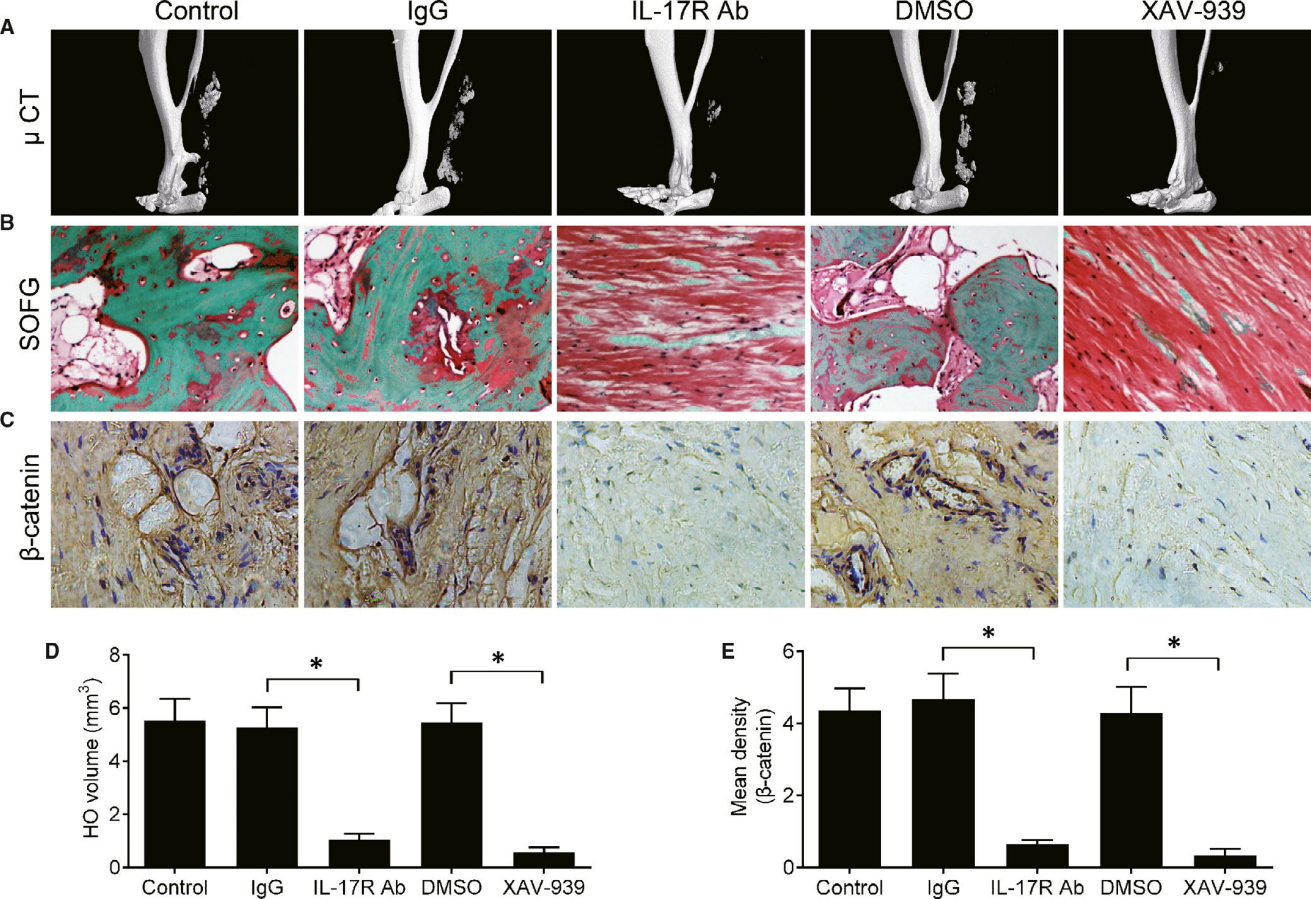
Inhibition of β‐catenin attenuates the formation of traumatic HO. The mice were injected with IL‐17 receptor antibody (IL‐17R Ab, 5 mg/kg bodyweight) or XAV‐939 (5 mg/kg bodyweight) at the Achilles tendon twice per week from day 0. IgG antibody and DMSO were used as negative controls. (A) μCT images of HO in the Achilles tendon 8 wk after operation. (B) Safranin O and Fast Green (SOFG) staining of HO tissue. (C) Immunohistochemical staining of β‐catenin in HO. (D) Quantitative analysis of HO volume by μCT. (E) Quantification of β‐catenin expression in HO. All data are shown as the mean ± SD. n = 6 per group. *P < .05

To evaluate the role of the β‐catenin signalling pathway in traumatic HO progression, the mice were injected with IL‐17R antibody or β‐catenin inhibitor XAV‐939 into the Achilles tendon twice per week. The μ‐CT results demonstrated that HO formation was abolished with injection of IL‐17R antibody 4 weeks post–Achilles tenotomy. Similar results were observed in the XAV‐939 injection group (Figure 4A,D). SOFG staining showed that new bone formation was significantly inhibited when the mice were treated with IL‐17R antibody or XAV‐939 (Figure 4B). In addition, the expression of β‐catenin was decreased in HO tissue after local injection with IL‐17R antibody or XAV‐939 (Figure 4C,E). Collectively, these results indicate that the IL‐17R/β‐catenin pathway is an important pathomechanism in traumatic HO.

### 3.5 IL‐17 enhances osteogenesis through the β‐catenin pathway

**FIGURE 5:**
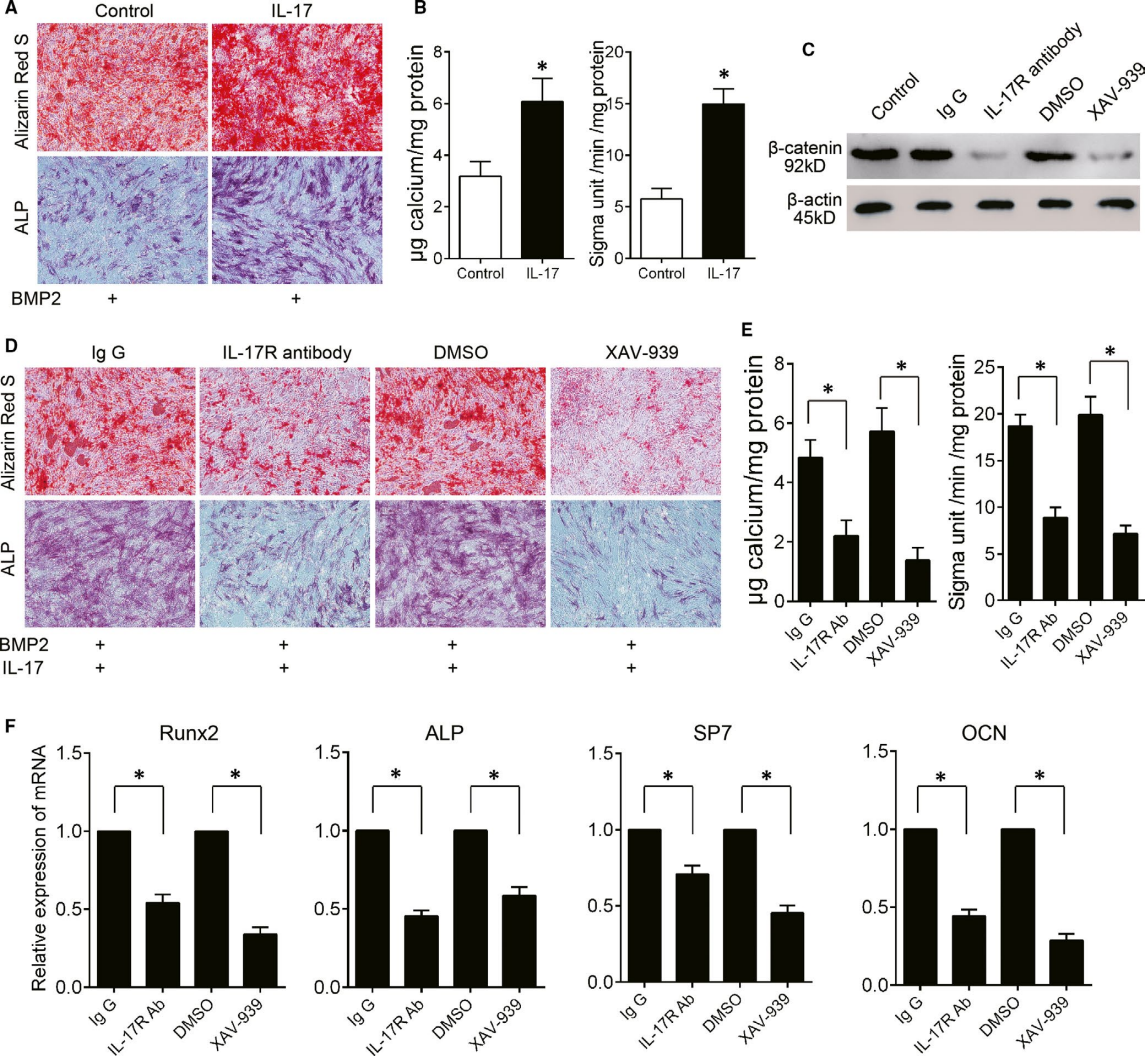
IL‐17 enhances osteogenesis through the β‐catenin pathway. (A) MSCs were cultured in BMP2 in the presence or absence of IL‐17. Alizarin red S staining was performed at week 2, and ALP staining was performed at week 1. (B) Quantification of calcium and ALP in MSCs treated with IL‐17. (C) MSCs were treated with IgG, IL‐17R antibody, DMSO or XAV‐939 for 24 h. β‐Catenin expression level was evaluated by Western blotting. (D) MSCs were cultured in the presence BMP2 and IL‐17 and treated with IL‐17R antibody or XAV‐939. IgG and DMSO were used as controls. Alizarin red S staining was performed at week 2, and ALP staining was performed at week 1. (E) Quantification of calcium and ALP in MSCs. (F) The mRNA expression levels of osteoblast genes in MSCs. n = 3. All data are shown as the mean ± SD

To examine the effects of IL‐17 on osteogenesis, MSCs were harvested from mouse bone marrow and induced by bone morphogenetic protein (BMP)‐2 in the presence or absence of IL‐17. The activity of alkaline phosphatase (ALP) and mineralization were up‐regulated by IL‐17 (Figure 5A,B). Then, the MSCs were treated with IL‐17R antibody or XAV‐939, and the Western blot results showed that β‐catenin activation was significantly inhibited (Figure 5C). To investigate whether IL‐17R/β‐catenin signalling plays an important role in osteogenesis, MSCs were treated with BMP‐2 and IL‐17 and exposed to IL‐17R antibody or XAV‐939. We found that both blockade of IL‐17R and β‐catenin signalling inhibited IL‐17‐induced ALP activation and mineralization deposition (Figure 5D,E). We analysed the effect of IL‐17R/β‐catenin signalling on the mRNA expression of osteoblast genes. The expression of Runx2, ALP, Sp7 and OCN, the essential transcription factors for osteogenesis, was significantly inhibited when IL‐17R or β‐catenin was blocked (Figure 5F). These data suggest that IL‐17 enhances MSC osteogenesis by activating the IL‐17R/β‐catenin pathway.

## 4 DISCUSSION

Heterotopic ossification is a pathological process that can occur as a result of trauma or as a consequence of genetic mutations. However, we still have limited knowledge about the exact pathogenesis of HO. Recent studies have shown that the immune system plays a pivotal role in the development of HO.^23^ In this study, we found that IL‐17 was highly induced after trauma. Injection of IL‐17 neutralizing antibody effectively attenuated HO progression in a traumatic HO animal model. IL‐17 promoted osteogenesis of MSCs harvested from injured tissues. We also found that IL‐17 enhanced ectopic bone formation by activating β‐catenin. These findings suggest that targeting IL‐17 in the early inflammation phase of HO development might be a possible solution for the dilemma.

IL‐17, produced mainly by TH_17_ cells, has been recognized as a proinflammatory cytokine that is involved in the induction of immune responses. Previously, it has been reported that IL‐17 plays an important role in the regulation of bone metabolism. However, its function has been mainly studied in the induction of osteoclastogenesis and development of bone resorption in arthritis.^24^ Other studies have demonstrated that IL‐17 induces osteoblast differentiation from human MSCs.^25^, ^26^ IL‐17 blocking antibody has been shown to be effective for spondyloarthropathy and psoriatic arthritis, chronic inflammatory diseases with excessive bone formation, indicating a positive effect of IL‐17 on bone formation.^27^, ^28^ These different results suggest that the effect of IL‐17 on osteoblast differentiation probably depends on the cell type.^24^ Our observations showed that at the early stage of HO, a large amount of IL‐17 was released into the tissue. Blockage of active IL‐17 by neutralizing antibody effectively retards HO progression in the animal model, suggesting that IL‐17 is an important triggering factor in HO. During the osteogenesis stage, osteoclasts are activated to degrade the bone mineral.^29^ We observed that the IL‐17 level and the number of TRAP^+^ cells were increased at the early stage of HO and then decreased at the later stage. These observations indicate that the interaction between osteoblasts and osteoclasts plays an important role in HO formation. When osteoblasts interact with osteoclasts, bone destruction occurs. However, when osteoclasts are not present, then the ectopic bone formation is observed.

IL‐17 binds to a heterodimeric receptor complex (IL‐17R) composed of IL‐17RA and IL‐17RC.^30^ IL‐17 signalling stimulates the transcription of inflammatory factors by activating the NF‐κB and MAPK pathways, including p38, ERK and JNK.^31^ It has been reported that IL‐17 promotes tissue regeneration and tumorigenesis by inducing ERK1/2 activation in the intestine.^32^ A recent study showed that IL‐17 up‐regulated β‐catenin expression in pulmonary hypertension.^33^ In the present study, we found that the expression of IL‐17 and IL‐17R was correlated with β‐catenin activation. Furthermore, our data showed that IL‐17 promoted osteogenic differentiation of MSCs via the β‐catenin pathway. Our experimental evidence from in vitro and in vivo studies demonstrated that blockade of IL‐17R or β‐catenin inhibited the formation of HO. These findings suggested that β‐catenin could be a functional target of IL‐17 and may mediate its regulatory role in HO formation.

It is still less known which progenitor cells are responsible for the initiation of traumatic HO. Several studies have demonstrated that vascular endothelial cells could be a candidate for the cellular origin of HO,^34^, ^35^ while other studies provide strong evidence that tissue‐resident MSCs or progenitor cell populations are the original cells in HO.^36^, ^37^ In trauma and wounds, MSCs in the tissue and circulation contribute to wound and fracture healing, which requires osteogenic differentiation of MSCs.^38^ It has been reported that the IL‐17 receptor (IL‐17R) can induce MSC differentiation into osteoblasts.^39^ Bone loss induced by ovariectomy was increased in mice deficient in IL‐17R.^40^ We demonstrated that the expression of IL‐17R was increased dramatically in the progression of HO. Moreover, blockade of IL‐17R suppressed the ectopic bone formation in vivo and osteogenic differentiation of isolated MSCs in vitro. These observations provide clinical insight into the contribution of IL‐17/IL‐17R pathway to the pathophysiological regulation of bone formation during traumatic HO progression.

The HO formation process has been anecdotally associated with enhanced osteoblast activity, and β‐catenin is one of the most important anabolic signalling pathways for osteoblast differentiation.^41^, ^42^ Aberrant Wnt/β‐catenin signalling activates BMP2, and coordination of the two pathways contributes to the development of HO in adrenal myelolipoma.^43^ Our previous study demonstrated that miR‐203 targeting Runx2 inhibits the formation of HO by suppressing β‐catenin.^21^ In the present study, we observed that β‐catenin is overactivated by IL‐17 in the mouse HO model.

Previous research has explored HO treatment by inhibiting inflammation with NSAIDs ^44^, ^45^ or BMP signalling inhibitors.^46^ However, there is no effective therapy for traumatic HO.^47^ In the present study, we demonstrated that injuries to the Achilles tendon reliably induced HO and increased active IL‐17 levels throughout HO progression. We found that the inhibition of IL‐17 activity successfully mitigates traumatic HO at different stages. We further provided evidence that IL‐17 induced the osteogenic differentiation of MSCs by activating β‐catenin and contributed to HO formation. Therefore, our findings suggest that inhibition of IL‐17 could be a new paradigm for the treatment of traumatic heterotopic ossifications.

## CONFLICT OF INTEREST

The authors declare no conflicts of interest.

## AUTHOR CONTRIBUTION

**Bing Tu:** Data curation (lead); Investigation (lead); Methodology (lead). **Bo Yu:** Funding acquisition (supporting); Investigation (equal); Project administration (equal). **Wei Wang:** Data curation (supporting); Formal analysis (supporting). **Juehong Li:** Investigation (supporting); Methodology (supporting); Project administration (supporting). **Feng Yuan:** Conceptualization (supporting); Data curation (supporting). **Jing Zhu:** Conceptualization (equal); Funding acquisition (supporting); Investigation (equal). **Cunyi Fan:** Conceptualization (equal); Funding acquisition (lead).
